# Factors associated with hostility among people living with HIV/AIDS in Northeast China: a cross-sectional study

**DOI:** 10.1186/s12889-019-7526-2

**Published:** 2019-08-29

**Authors:** Miaomiao Zhao, Baohua Liu, Tong Zheng, Jiao Xu, Yanhua Hao, Jiahui Wang, Xin Zhang, Wanling Nie, Chao Wang, Fuxiang Wang, Mingli Jiao, Qunhong Wu, Libo Liang

**Affiliations:** 1 Department of Social Medicine, School of Health Management, Harbin Medical University, 157 Baojian Road, Nangang District, Harbin, 150081 Heilongjiang China; 2 Department of Health Management, School of Public Health, Nantong University, 9 Seyuan Road, Chongchuan District, Nantong, 226019 Jiangsu China; 3 The Second Affiliated Hospital of Harbin Medical University, 247 Xuefu Road, Nangang District, Harbin, 150001 Heilongjiang China; 4 The First Affiliated Hospital of Harbin Medical University, 23 Youzheng Road, Nangang District, Harbin, 150001 Heilongjiang China; 5 The Third People’s Hospital of Shenzhen, 29 Bulan Road, Longgang District, Shenzhen, 518100 China

**Keywords:** HIV/AIDS, Hostility, Mental health, Depression

## Abstract

**Background:**

Hostility can result in negative outcomes in people living with HIV/AIDS (PLWHA); however, previous research on this topic is far from adequate. To contribute to existing knowledge on this aspect of PLWHA, the current study examined the prevalence of hostility and its potential influencing factors among PLWHA.

**Methods:**

A cross-sectional questionnaire survey was undertaken on 218 HIV patients in Heilongjiang Province of China between March and August in 2013. A multiple logistic regression analysis was performed to identify factors associated with hostility.

**Results:**

The prevalence of hostility was 17.0% among the participants. The three most alarming types of hostility included desiring to kill the person who infected them, blaming the infection on the society, and abandoning themselves to despair. A multiple logistic regression model identified that depression (OR = 3.845, 95% CI = 1.309–9.229), perceived stigma (OR = 3.281, 95% CI = 1.109–7.711), and fear of dying (OR = 2.710, 95% CI = 1.068–6.881) were the risk factors for hostility, while higher levels of trust-in-doctor (OR = 0.176, 95% CI = 0.060–0.517) and per capita household income (OR = 0.344, 95% CI = 0.119–0.991) were protective factors.

**Conclusions:**

Our findings highlight the prominent influence of psychological, healthcare, and social factors on hostility among PLWHA. Interventions specifically targeted to reduce hostility should be provided, including incorporating psychological service into HIV management guidelines, enhancing PLWHA’s trust-in-doctor, establishing comprehensive services for PLWHA, reducing the social stigma against PLWHA, and paying more attention to PLWHA with financial problems. These interventions may improve the management and control of HIV/AIDS.

## Background

Acquired immune deficiency syndrome (AIDS) is a spectrum of conditions caused by human immunodeficiency virus (HIV), and is considered one of the most serious health and development issues in the world [1]. According to the World Health Organization (WHO), nearly 36.9 million people were living with HIV/AIDS in 2017, including 1.8 million new cases [2]. Despite substantial efforts in tackling the HIV/AIDS epidemic in China, the total number of people living with HIV/AIDS (PLWHA) is still increasing, which is a fact backed up by evidence: the number of PLWHA nearly doubled between 2012 and 2017, going from 385,817 to 758,610 [3, 4]. This increasing number of HIV cases poses huge challenges to China concerning treatment, care, and support for PLWHA.

The advancements in antiretroviral therapies (ART) have greatly decreased the mortality rates among PLWHA, as well as improved their survival time, which has transformed HIV/AIDS from a terminal illness to a chronic disease [5]. Consequently, PLWHA face more psychological challenges since they need to cope with HIV/AIDS during their extended lifetime [6]. For example, since HIV/AIDS is an incurable disease often accompanied by severe symptoms, PLWHA are faced with high risk of death. Further, PLWHA are easily rejected and stigmatized owing to the misconceptions about HIV transmission and disapproved risk behavior among the infected groups such as men who have unprotected sex with men, sex workers and injection drug users [7]. Additionally, PLWHA are more likely to be exposed to a wide variety of stressful situations, including having somatic symptoms, receiving long-term medical treatments, as well as experiencing medication side effects, marital conflicts, job loss and financial problems [8–10]. Such factors are associated with an increased risk of developing psychological disorders such as depression, anxiety, guilt and loneliness [11–13]. Thus, in the absence of support from their social environment or psychological services, PLWHA might turn these internalized negative emotions into hostility towards society [14]. Previous research had corroborated that PLWHA experienced a higher risk of hostility, compared to healthy individuals [15, 16].

Hostility can be conceptualized as a negative attitude towards the outside world [17, 18], and its impact on a person’s life is multifaceted. Previous studies showed that hostility could impact C-reactive protein interleukin, and proinflammatory cytokines to cause inflammation [19–21], which may worsen the physical health of PLWHA. Further, hostility was associated with engagement in risk behaviors, such as injection drug use, unprotected sex and suicide attempts [22, 23]. In addition, hostility could also lead to dire consequences for society because it had relations with aggressive, vengeful, and antisocial behaviors [24, 25]. In summary, hostility is a potential threat to the health of PLWHA, and to HIV/AIDS control and social safety.

Prior studies showed that hostility can be changed both through external events and internally adjustments related to somatic or psychiatric symptoms [18]. Therefore, exploring potential factors associated with hostility can play a pivotal role in reducing hostility among PLWHA. In this regard, implementing interventions associated with these factors may be important not only for improving the health of PLWHA, but also for controlling the harmful consequences for society. Despite these potential benefits, there is limited research examining the influencing factors of hostility among PLWHA. To our knowledge, only one previous study had explored the association between perceived stigma and hostility among men who have sex with men (MSM) with HIV/AIDS and found that the perceived stigma predicted a higher level of hostility [26]. Thus, more researches are needed on the examination of hostility and its influencing factors among PLWHA.

In this study, we aimed to assess the prevalence of hostility among PLWHA‚ and to explore the multidimensional factors (including sociodemographic characteristics, disease related characteristics, self-reported physical health status, psychological factors, healthcare service factors and social factors) that may influence hostility. Our results could be used as evidence by policymakers and health service providers to improve HIV/AIDS care and intervention programs.

## Method

### Study population and data collection

We conducted a cross-sectional survey among PLWHA in an HIV-designated hospital in Heilongjiang Province between March and August in 2013. Chinese patients who were aged 18 years and above, and diagnosed with an HIV infection were included in this study. During patients’ regular hospital visits, all eligible patients were briefed by their physicians on the purpose and value of this investigation. The ones who agreed to participate were referred to the researchers by their physicians. Then, the researchers gave each patient a full explanation regarding the procedures of the study. Voluntary participation was emphasized throughout the study. After signing written informed consent, the participants were asked to complete a self-administrated questionnaire. To ensure quality, a researcher was present during the survey and would provide assistance upon participants’ request. The researchers who participated in this process were from Social Medicine Department of Harbin Medical University and had received training on HIV/AIDS related research. All personal identifiers were removed from the questionnaires to ensure confidentiality.

In total, 241 patients were approached during the survey, and all of them agreed to participate in the survey. However, 14 patients dropped out during the investigation because they lacked time to complete this long questionnaire. Afterwards, During subsequent data cleaning process, 9 patients were further excluded owing to missing data (including 3 non-Han ethnic, 2 illiterates and 4 other participants, who were excluded from the sample for further statistical analysis). Finally, 218 participants were involved in this study.

### Variables

#### Dependent variable

Based on previous studies, we believe that the psychological reactions of patients with HIV infection differ from those of other patients or the general population: apart from severe physical health damage, PLWHA also experience stigma and social exclusion. Thus, in this study, we used a hostility questionnaire specifically developed for PLWHA by Meng [27] to estimate participants’ level of hostility. The questionnaire consists of six items including: “Do you abandon yourself to despair?”, “Do you feel like you hate the outside world?”, “Do you have a desire to kill the person who infected you?”, “Do you blame the infection on society and others?”, “Do you feel abandoned by society?”, “Do you have the urge to retaliate against society?” Each item was scored on a scale ranging from 1 (never) to 5 (all of the time). The scores of all the six items were taken into average, and a higher average score indicated a higher level of hostility. An average score greater than 2 was drawn as the demarcation criterion for having hostility. The Cronbach’s α in the present study was 0.92, indicating a high internal consistency.

#### Independent variable

Sociodemographic data of PLWHA were collected (see Table 1), including gender, age (< 30, 31–39, 40–49, or ≥ 50 years), marital status (married/cohabitating or others), educational status (junior high school and below, senior high school, college, or undergraduate and above) and per capita household income (≤2000 or > 2000 CNY per month).

**Table 1:** Participants’ characteristics and their associations with hostility

	Total, N (%)	Hostility		p
		Yes, n (%)	No, n (%)	
Sociodemographic characteristics				
Sex				0.626^a^
Male	210 (96.3)	35 (16.7)	175 (83.3)	
Female	8 (3.7)	2 (25.0)	6 (75.0)	
Age (years)				0.400
< 30	69 (31.7)	12 (17.4)	57 (82.6)	
30–39	64 (29.4)	7 (10.9)	57 (89.1)	
40–49	53 (24.3)	12 (22.6)	41 (77.4)	
≥50	32 (14.7)	6 (18.8)	26 (81.3)	
Marital status				0.849
Married or cohabitating	62 (28.4)	11 (17.7)	51 (82.3)	
Other	156 (71.6)	26 (16.7)	130 (83.3)	
Education				0.256
Junior high school and below	53 (24.3)	13 (24.5)	40 (77.5)	
Senior high school	52 (23.9)	9 (17.3)	43 (82.7)	
College	47 (21.6)	8 (17.0)	39 (83.0)	
Undergraduate and above	66 (30.3)	7 (10.6)	59 (89.4)	
Per capita household income (CNY)				**0.005**
> 2000	105 (48.2)	10 (9.5)	95 (90.5)	
≤ 2000	113 (51.8)	27 (23.9)	86 (76.1)	
Disease related characteristics				
Infection routes				0.999
Homosexual contact	149 (68.3)	25 (16.8)	124 (83.2)	
Heterosexual contact	28 (12.8)	5 (17.9)	23 (82.1)	
Blood	17 (7.8)	3 (17.6)	14 (82.4)	
Others	24 (11.0)	4 (16.7)	20 (83.3)	
Infection duration (years)				0.796
≤ 1	80 (36.9)	11 (13.8)	69 (86.3)	
1 < X ≤ 3	82 (37.8)	16 (19.5)	66 (80.5)	
> 3	55 (25.3)	10 (18.2)	45 (81.8)	
CD4 + cell (cells/mm3)				0.669
≤ 200	83 (38.1)	12 (14.5)	71 (85.5)	
200–500	117 (53.7)	21 (17.9)	96 (82.1)	
> 500	18 (8.3)	4 (22.2)	14 (77.8)	
Self-reported physical health status				**0.004**
High	123 (56.4)	13 (10.6)	110 (89.4)	
Low	95 (43.6)	24 (25.3)	71 (74.7)	
Psychological factors				
Depression				**0.000**
Yes	104 (47.7)	29 (27.9)	75 (72.1)	
No	114 (52.3)	8 (7.0)	106 (93.0)	
Anxiety				**0.007**
Yes	103 (47.2)	25 (24.3)	78 (75.7)	
No	115 (52.8)	12 (10.4)	103 (89.6)	
Fear of dying				**0.001**
Yes	77 (35.3)	20 (26.0)	57 (74.0)	
No	141 (64.7)	17 (12.1)	124 (87.9)	
Healthcare service factors				
Satisfaction with healthcare				**0.026**
Yes	146 (67.6)	18 (12.3)	128 (87.7)	
No	70 (32.4)	17 (24.3)	53 (75.5)	
Trust-in-doctor				**0.009**
High	119 (54.6)	13 (10.9)	106 (89.1)	
Low	99 (45.5)	24 (24.2)	75 (75.8)	
Social factors				
Family and social support				
High	134 (61.5)	17 (12.7)	117 (87.3)	**0.033**
Low	84 (38.5)	20 (23.8)	64 (76.2)	
Perceived stigma				**0.014**
High	138 (63.3)	30 (21.7)	108 (78.3)	
Low	80 (36.7)	7 (8.8)	73 (91.3)	

^a^Fisher’s exact test

Disease related characteristics of the participants were also collected (see Table 1), including infection route (homosexual contact, heterosexual contact, blood, or others), infection duration (≤1, 1–3 and > 3 years), and CD4 + cell (≤200, 200–500 or > 500 cells/mm^3^).

To measure the self-reported physical health status of PLWHA, we used the physical health domain of the WHOQOL-HIV-BREF questionnaire, which included four items (pain/discomfort, energy, symptoms and sleep), with a higher score indicating a better physical health status [28]. The Chinese WHOQOL-HIV-BREF had been validated in previous studies for Chinese PLWHA [29, 30]. The Cronbach’s α of these four items was 0.753 in this study. We adopted the mean score as the cut-off score for dichotomization of the physical health status (high level and low level).

Psychosocial factors included depression, anxiety, and a fear of dying. Depression was assessed using Burns Depression Checklist (BDC), consisting of 15 items rated on a scale from 0 (not at all) to 3 (a lot). The total score ranged from 0 to 45, with a higher score indicating more severe depression [31]. This scale had been previously validated in China [32]. Individuals with a total score equal to or greater than 11 were considered to have depression [33].The Cronbach’s α of the BDC scale was 0.96 in the current study. Zung Self-Rating Anxiety Scale (SAS) was used to evaluate the level of anxiety symptoms [34]. It consisted of 20 items rated on a scale ranging from 1 (a little of the time) to 4 (most of the time). The total score ranged from 20 to 80, which was then transformed to a SAS Index score (raw SAS score multiplied by 1.25). Individuals with a SAS Index score equal to or greater than 50 were considered to have anxiety, and this scale had been widely used and validated in China [35, 36]. The Cronbach’s α of the SAS scale was 0.89 in this study. Finally, an individual’s fear of dying was measured using one item: “During the disease, did you experience fear of dying?” Participants answered Yes (=1) or No (=0) to this question.

Healthcare service factors included healthcare satisfaction and trust-in-doctor. Health care satisfaction was measured using the question “Are you satisfied with the health care you received?” Participants answered based on a 5-point Likert scale ranging from 1 (strongly dissatisfied) to 5 (strongly satisfied). For data analysis, responses were recoded as: “Not Satisfied” (ranging from 1 to 3) and “Satisfied” (ranging from 4 to 5). To assess patient’s trust-in-doctor, the Wake Forest Physician Trust Scale (WFPTS) was used [37]. A previous study showed that the Chinese version of the WFPTS had good reliability and validity [38]. This instrument contained 10 items, which were rated on a 5-point Likert scale ranging from 1 (strongly disagree) to 5 (strongly agree), with a higher score indicating a greater trust-in-doctor. We adopted the mean score as the cut-off score for dichotomization of participants’ trust (high level and low level). The Cronbach’s α of this scale was 0.79 in this study.

Social factors included family and social support, and perceived stigma. The Chinese Family and Social Support questionnaire for PLWHA was used to measure the frequency of family and social support based on a 5-point Likert scale ranging from 1 (none of the time) to 5 (all the time), with a higher score indicating a higher level of family and social support for PLWHA [27]. We adopted the mean as the cut-off score for dichotomization of support (high level and low level). In this study, the Cronbach’s α of this scale was 0.87. To assess perceived stigma, the simplified version of Berger HIV Stigma scale was used, with a higher score indicating a higher level of perceived HIV stigma [27]. This scale had been validated in a previous study for Chinese PLWHA [39]. The mean score was adopted as the cut-off score for dichotomization of stigma (high level and low level). This scale had a high internal consistency (Cronbach’s α = 0.90) in this study.

### Statistical analyses

First, we used descriptive statistics to describe participants’ basic characteristics and the prevalence of hostility among PLWHA. Chi-square tests were performed to analyze the differences in hostility across different levels of independent variables. Then, a multiple logistic regression was performed to examine the associations between hostility and its influencing factors among PLWHA (method = ENTER). The independent variables that were found to be associated with hostility in the Chi-square tests (*p* < 0.05) were entered into the logistic regression, and sociodemographic characteristics were also controlled. All data were analyzed using SPSS 21.0, and *p* < 0 .05 was considered significant.

## Results

### Participants’ characteristics

Of the 218 participants, the majority (96.3%) were male and most (85.3%) were 49 years or below. About three-fourths of the participants had finished high school (75.7%). Less than one-third (30.3%) were currently married, and less than half (48.2%) had a per capita household income over 2000 CNY per month.

The main route of infection was sexual contact (81.2%). Most participants (91.7%) had CD4 count less than or equal to 500 cells/mm^3^, and the average duration of the infection was 2.2 years. More than half (56.4%) of the participants reported high level of physical health. In addition, 47.7 and 47.2% of the participants suffered from depression and anxiety respectively, and 35.3% reported fear of dying. More than half of the participants reported a high level of trust-in-doctor, family and social support, and perceived stigma (54.6, 61.5 and 63.3%, respectively) (see Table 1).

### Prevalence of hostility among PLWHA

Of the 218 PLWHA, 37 (17.0%) reported hostility towards society. Chi-square tests showed that hostility was significantly positively associated with depression, anxiety, fear of dying and perceived stigma, and negatively associated with per capita household income, self-reported physical health status, satisfaction with healthcare, trust-in-doctor, family and social support (*p* < 0.05). Participants who had a higher per capita household income, higher level of trust-in-doctor and family and social support, better physical health status, and were satisfied with healthcare were less likely to develop hostility. While those who had depression, anxiety, fear of dying, and higher level of perceived stigma were more likely to develop hostility (see Table 1).

### Comparison of the hostility items scores between hostile and non-hostile PLWHA

**Fig. 1:**
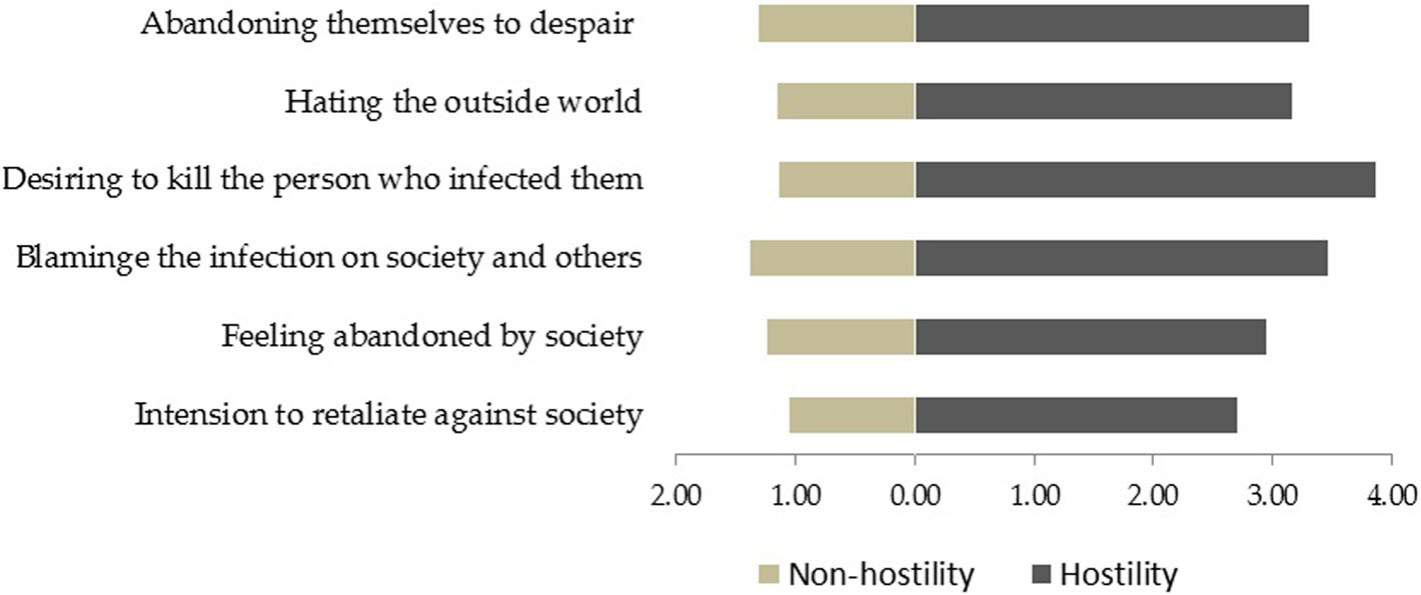
Scores of specific hostility items among hostile and non-hostile PLWHA

We compared the mean scores for each item in the hostility questionnaire between the hostile and non-hostile PLWHA. The results showed that mean scores for all the items among the hostile PLWHA were significantly higher than those among the non-hostile PLWHA (Wilcoxon’s rank sum test). Of all the items, the hostile group had the highest mean score for “desiring to kill the person who infected them” (3.86 ± 1.11), followed by “blaming the infection on society and others” (3.46 ± 1.36), “abandoning themselves to despair” (3.30 ± 1.29) and “hating the outside world” (3.16 ± 1.19), whereas the non- hostile group’s mean scores were less than 2 for all items. Fortunately, among both groups, the mean score for “intension to retaliate against society” were the lowest (see Fig. 1).

### Factors associated with hostility in logistic regression

**Fig. 2:**
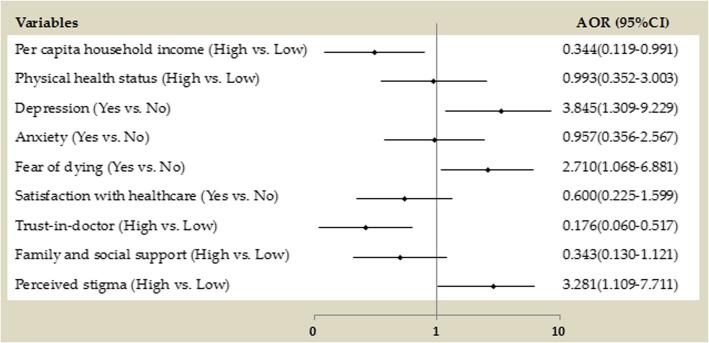
Factors associated with hostility in logistic regression model

Independent variables that were significantly associated with hostility in the univariate analysis were entered into a logistic regression model. In addition, sociodemographic characteristics including sex, age, marital status and education were also controlled in the analyses (these four control variables were not significant; for brevity, we shall not present the results of control variables). Results showed that five factors were significantly associated with hostility among PLWHA. PLWHA who experienced depression were 3.845 times (95% CI 1.309 to 9.229) more likely to develop hostility than those without depression. Further, PLWHA who reported a high level of perceived stigma were 3.281 times (95% CI 1.109 to 7.711) more likely to develop hostility than those who reported a low level of perceived stigma. PLWHA who reported a fear of dying were 2.710 times (95% CI 1.068 to 6.881) more likely to develop hostility than those who did not. In addition, a high level of per capita household income (OR = 0.344, 95% CI 0.119 to 0.991) and trust-in-doctor (OR = 0.176, 95% CI 0.060 to 0.517) could significantly reduce the risk for hostility (see Fig. 2).

## Discussion

This study contributed to the existing knowledge on hostility among PLWHA and its influencing factors. Results showed that 17.0% of the participants experienced hostility towards society. Further, this rate was higher than that in a previous study conducted in Nigeria, which showed a prevalence rate of hostility was 15.0% among PLWHA [40]. PLWHA who were depressed, had a fear of dying, and had a higher level of perceived HIV stigma were at higher risk for hostility, while those who had a higher level of trust-in-doctor and per capita household income were at lower risk. This finding is important for health professionals in planning targeted intervention strategies to help mitigate hostility among PLWHA.

Regarding the specific types of hostility, the three most alarming types included “desiring to kill the person who infected them”, “blaming the infection on society and others”, and “abandoning themselves to despair”. It is noteworthy that the mean scores for “desiring to kill the person who infected them” and “blaming the infection on society and others”— reflecting how PLWHA hold external and other factors such as the society and other person who infected them responsible for their infection and disease —were nearly 4 (in a maximum of 5). HIV diagnosis could be extremely distressing [41], and that it was common for patients to not accept the fact of their infection, to consider that they are victims, and to further blame other people or society for their situation [42]. Cherilyn and Clement indicated that PLWHA who blamed others for their HIV infection might also have the feeling of inequity or anger (i.e., they believed that they did not deserve to be infected), and if they were unable to redress the situation, they could transfer these feelings directly to the person who infected them, or even to those unrelated with the infection [43, 44]. This might pose a threat to the safety of the society, and warrant greater attention and further investigation.

In this study, depression was the strongest variable associated with hostility. PLWHA with depression were nearly four times more likely to develop hostility, compared to those without depression. Indeed, depression was considered as one of the most common mental health conditions experienced by PLWHA [45, 46], and this study partially corroborated to that notion as data analysis showed that 47.7% of PLWHA suffered from depression. Liu‘s study showed that the prevalence of depression might be even higher (60.6%) among Chinese PLWHA [47]. Depression might be related to hostility for the following reasons. First, depression is commonly accompanied by other symptoms such as anger or irritability [48–50]; a persistent or frequent feeling of anger might be highly associated with or even be regarded as hostility [51]. Second, it is possible that depressed individuals encounter more barriers in achieving healthy emotion regulation, which could evoke more negative emotions [52]. Third, depression also could serve as a moderating factor for social disorder and hostility [25]. Thus, successful management of depression is a key to mitigating hostility among PLWHA. However, the majority of services provided by *China Four Frees and One Care Program* focus primarily on physical rather than psychological symptoms; indeed, PLWHA are not adequately screened or treated for psychological problems. Therefore, development of regular psychological healthcare services for PLWHA is urgently needed. This action may have additional benefits that go beyond reducing hostility—it may help patients better adhere to their ART medications and improve treatment retention, which are critical to achieving the WHO’s 90–90-90 global targets.

In this study, as many as 63.3% of the participants reported a high level of perceived stigma, indicating that despite many years of effort and campaign for anti-discrimination, social stigma against PLWHA is still a serious concern in China. PLWHA are highly discriminated against in China, partly because the HIV-related risk behaviors such as homosexuality, commercial sex or drug abuse are not accepted by sociocultural norms [53, 54]. Supporting this assumption, a study conducted in China reported that over 90% of PLWHA perceived HIV-related stigma [55]. Widespread stigmatization and discrimination against PLWHA might in turn trigger PLWHA to be dissatisfied with or resentful against society [26, 56]. In this study, we found that perceived stigma was the second highest contributing factor for hostility. PLWHA with a higher level of perceived stigma were 3.281 times more likely to develop hostility. Currently, advancements in public health programs to address stigma have been comparatively slow and unsystematic in China [57]. Thus, media publicity and educational strategies aimed at converting and mitigating misunderstanding and fear of HIV/AIDS among the public could help reduce social stigma against PLWHA, thereby reducing hostility among PLWHA.

In this study, one-third of PLWHA reported a fear of dying, which was also significantly associated with hostility. PLWHA with a fear of dying were 2.710 times more likely to develop hostility than those without this fear. As there is currently no definite cure for HIV/AIDS, it is not surprising that it evokes the fear of dying. Fear of dying not only revealed psychological stress on PLWHA [58], but also predicted difficulties in coping with stressful events [58, 59]. These facts might explain the association between fear of dying and a higher risk for hostility; however, further analyses are needed to confirm this relationship.

Regarding the healthcare service related factors, a higher level of trust-in-doctor stood out as an important protective factor for hostility. PLWHA with higher trust in their doctors were less likely to experience hostility, compared to those with lower trust. Due to discrimination and stigma, it could be particularly difficult for PLWHA to disclose their infection to friends, family, and sex partners [60, 61]. Additionally, it had been reported that non-disclosure was associated with lower levels of social support [62]. In this context, PLWHA usually regard their doctors whom they can discuss their situation with as a crucial part of their social support networks [63]. Therefore, a trust-worthy relationship with doctors might be an important supportive factor for PLWHA’s psychological health and clinical treatment. Nevertheless, this study found that 45.3% of the participants did not have a high level of trust-in-doctor. This might be partly associated with deteriorative patient-doctor relationships in China. Moreover, discriminatory attitudes and lack of willingness to interact with AIDS patients displayed by Chinese health professionals might also deteriorate patients’ trust [64]. Nowadays, the majority of providers of HIV-related services in China are clinicians who have not been trained in the provision of psychological interventions [65], which may directly interfere with their ability to meet the multidimensional needs of PLWHA. Based on these facts, there is an urgent need to establish comprehensive treatment and care services including clinical treatment, psychological care and social support for PLWHA in China. This could be potentially valuable for reducing hostility among PLWHA.

Further into the protective factors for hostility, among the sociodemographic characteristics, higher per capita household income was a significant protective factor. Household income plays an important part in the economic and social stability of PLWHA [66]. However, HIV infection could lead to a decrease in household income, since patients cannot work as much as before, and some might have to quit or change their jobs [67, 68]. A previous study showed that household income reduced on average by 16% after a member of the household was infected with HIV [69]. In our study, more than half the participants had a lower household income (less than 2000 CNY per capita monthly) and more than half of the participants were at the age stage of earning the main salary in their family (30–49 years). However, the possibility of failing to provide for their family due to their reduced work ability may place great psychological burden on PLWHA, which may, in turn, increase their risk of hostility towards society. Policies aimed at maintaining the household income of PLWHA (e.g., by providing appropriate re-employment chances) should also be a priority in China.

Our study has several limitations. First, the data were collected through a self-reported questionnaire; therefore, recall bias and social desirability bias may exist. Second, as it was a cross-sectional study, we could not establish causal relationships based on the results. Third, our sample included only people who agreed to participate in this study, thus, findings in this sample might differ from those in PLWHA who refused. Fourth, the generalizability of the findings was limited because after the data cleaning process, we did not include non-Han and non-illiteracy PLWHA who failed to complete the questionnaire and this study was only conducted in one HIV-designated hospital in Heilongjiang. Further studies with a larger and more diverse sample and those that thoroughly analyze the mechanisms of various factors influencing hostility among PLWHA, are needed.

## Conclusions

The prevalence of hostility among PLWHA in the present study was 17.0%. Further, depression, perceived stigma, fear of dying, trust-in-doctor, and per capita household income were associated with hostility. Our findings highlighted the prominent influence of psychological, healthcare, and social factors on hostility among PLWHA, which are important for developing more targeted intervention strategies for reducing hostility among PLWHA. First, the psychological component of PLWHA should be incorporated into HIV/AIDS management guidelines, aiming at providing screening and appropriate care for psychological disorders, and consequently reducing PLWHA’s negative beliefs about the disease and society. Second, to enhance PLWHA’s trust-in-doctor, in addition to provide better communication and more humanistic care, healthcare professionals also should be provided with specific educational programs to help improve their attitudes, knowledge and practices regarding HIV/AIDS. Third, there is an urgent need to establish comprehensive services including clinical treatment, psychological care and social support for PLWHA in China. Fourth, social interventions should focus on changing the social stigma against PLWHA by raising disease awareness, as well as providing more financial and medical assistance to PLWHA with financial problems.

## Data Availability

The datasets are available from the corresponding author on reasonable request.

## Abbreviations

AIDS: Acquired Immunodeficiency Syndrome
ART: Antiretroviral Therapy
BDC: Burns Depression Checklist
CNY: Chinese Yuan
HIV: Human Immunodeficiency Virus
MSM: Men Who Have Sex with Men
PLWHA: People Living With HIV/AIDS
SAS: Zung Self-Rating Anxiety Scale
WFPTS: The Wake Forest Physician Trust Scale
WHO: World Health Organization
